# Identification of Novel Viruses in *Amblyomma americanum*, *Dermacentor variabilis*, and *Ixodes scapularis* Ticks

**DOI:** 10.1128/mSphere.00614-17

**Published:** 2018-03-07

**Authors:** Rafal Tokarz, Stephen Sameroff, Teresa Tagliafierro, Komal Jain, Simon H. Williams, D. Moses Cucura, Ilia Rochlin, Javier Monzon, Giovanna Carpi, Danielle Tufts, Maria Diuk-Wasser, Jory Brinkerhoff, W. Ian Lipkin

**Affiliations:** a Center for Infection and Immunity, Mailman School of Public Health, Columbia University, New York, New York, USA; b Division of Vector Control, Suffolk County Department of Public Works, Yaphank, New York, USA; c Natural Science Division, Pepperdine University, Malibu, California, USA; d Department of Molecular Microbiology and Immunology, Johns Hopkins Bloomberg School of Public Health, Baltimore, Maryland, USA; e Department of Ecology, Evolution and Environmental Biology, Columbia University, New York, New York, USA; f Department of Biology, University of Richmond, Richmond, Virginia, USA; University of Pittsburgh

**Keywords:** *I. scapularis*, ticks, virome

## Abstract

Ticks carry a wide range of known human and animal pathogens and are postulated to carry others with the potential to cause disease. Here we report a discovery effort wherein unbiased high-throughput sequencing was used to characterize the virome of 2,021 ticks, including *Ixodes scapularis* (*n =* 1,138), *Amblyomma americanum* (*n =* 720), and *Dermacentor variabilis* (*n =* 163), collected in New York, Connecticut, and Virginia in 2015 and 2016. We identified 33 viruses, including 24 putative novel viral species. The most frequently detected viruses were phylogenetically related to members of the *Bunyaviridae* and *Rhabdoviridae* families, as well as the recently proposed *Chuviridae*. Our work expands our understanding of tick viromes and underscores the high viral diversity that is present in ticks.

**IMPORTANCE** The incidence of tick-borne disease is increasing, driven by rapid geographical expansion of ticks and the discovery of new tick-associated pathogens. The examination of the tick microbiome is essential in order to understand the relationship between microbes and their tick hosts and to facilitate the identification of new tick-borne pathogens. Genomic analyses using unbiased high-throughput sequencing platforms have proven valuable for investigations of tick bacterial diversity, but the examination of tick viromes has historically not been well explored. By performing a comprehensive virome analysis of the three primary tick species associated with human disease in the United States, we gained substantial insight into tick virome diversity and can begin to assess a potential role of these viruses in the tick life cycle.

## Abstract (precis)

The incidence of tick-borne disease is increasing, driven by rapid geographical expansion of ticks and the discovery of new tick-associated pathogens. The examination of the tick microbiome is essential in order to understand the relationship between microbes and their tick hosts and to facilitate the identification of new tick-borne pathogens. Genomic analyses using unbiased high-throughput sequencing platforms have proven valuable for investigations of tick bacterial diversity, but the examination of tick viromes has historically not been well explored. By performing a comprehensive virome analysis of the three primary tick species associated with human disease in the United States, we gained substantial insight into tick virome diversity and can begin to assess a potential role of these viruses in the tick life cycle.

## INTRODUCTION

Ticks (order Ixodida) are hematophagous arthropods that are second only to mosquitos as vectors of human pathogens worldwide. The ability of ticks to transmit a wide range of microbial pathogens, combined with their promiscuous feeding and geographical range expansion, makes them a substantial threat to animal and human health (1). Several recent studies have described novel tick-transmitted pathogens, suggesting that the full spectrum of tick-borne pathogens has not been identified (2–5).

Genomic analyses using unbiased high-throughput sequencing (UHTS) platforms have proven valuable for investigations of microbially diverse populations, and recently these analyses have been extended to include ticks. The characterization of the tick microbiome is fundamental to understanding the relationship between microbes and their tick hosts and may facilitate the discovery of novel pathogens. UHTS has enabled the examination of tick viromes, a component of the tick microbiome that historically has not been well explored (6–12). In 2014, we reported the discovery of nine new viruses that were detected in *Ixodes scapularis* (the blacklegged tick), *Dermacentor variabilis* (the American dog tick), and *Amblyomma americanum* (the lone star tick) collected at a single site in New York State (6, 13). Several of these viruses were also identified in a recent study of *I. scapularis* ticks from Pennsylvania (14). These investigations have provided an insight into the breadth of viral genetic diversity found in ticks in the northeastern United States. In this study, we expanded on our previous work by examining a greater population of these three tick species collected from multiple geographically diverse sites in New York, Connecticut, and Virginia.

## RESULTS

**FIG 1:**
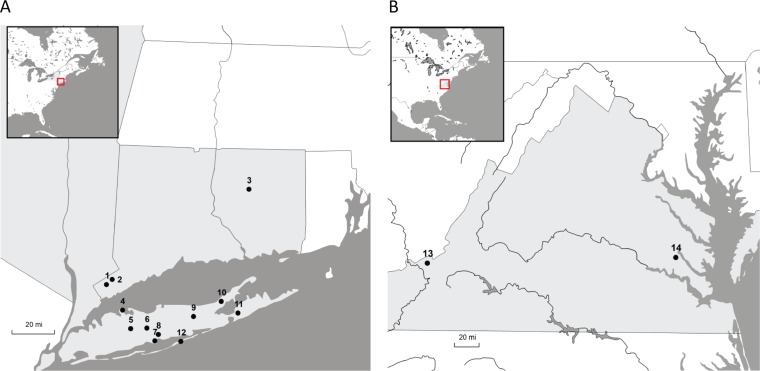
Tick collection sites. Each dot represents a different site, with panel A showing the sites in Connecticut (labeled 1 to 3) and Long Island, NY (labeled 4 to 12), and panel B showing Virginia sites. The numbers correspond to the following locations: 1, Bobcock Nature Preserve; 2, Mianus Nature Preserve; 3, Fifty-Foot Cliff Park; 4, Caumsett State Park; 5, Brentwood; 6, West Hills County Park; 7, Heckscher State Park; 8, Connetiquot State Park; 9, Manorville; 10, Laurel Lake; 11, Tuckahoe Hill Preserve; 12, Fire Island; 13, Giles County; 14, New Kent County. Maps were created using StepMap.

We analyzed a total of 2,021 ticks collected at nine sites on Long Island, NY, three sites in Connecticut, and two sites in Virginia (Fig. 1; Table 1). The ticks were combined into 91 pools, including 39 pools of *I. scapularis* adults (879 ticks), 10 pools of *I. scapularis* nymphs (259 ticks), 34 pools of *A. americanum* adults (720 ticks), and 8 pools of *D. variabilis* adults (163 ticks) (Table 2). We identified sequences of 33 viruses. These viruses included 24 putative novel species and 9 previously characterized viruses (Table 3). The largest number of viruses was detected in *I. scapularis*-derived pools, where 21 viruses were identified, including 14 novel species. All 21 viruses were present in adults; 5 of these viruses were also present in nymphs. *A. americanum* pools contained 6 novel viruses, and 6 viruses were present in *D. variabilis* pools (including 4 novel species). Each virus identified in this study was associated with only a single tick species (Table 3).

**TABLE 1:** Distribution of tick pools from each collection site

Location^a^	Tick species	No. of pools
1. Babcock Nature Preserve, CT	*I. scapularis*	3
2. Mianus River State Park, CT	*I. scapularis*	5
3. Fifty Foot Cliff Park, CT	*I. scapularis* (nymphs)	10
4. Caumsett State Park, NY	*I. scapularis*	8
5. Brentwood, NY	*D. variabilis*	4
6. West Hills County Park, NY	*A. americanum*	3
7. Heckscher State Park, NY	*I. scapularis*	7
	*A. americanum*	4
	*D. variabilis*	2
8. Connetiquot State Park, NY	*I. scapularis*	7
	*A. americanum*	10
9. Manorville, NY	*I. scapularis*	4
	*A. americanum*	13
	*D. variabilis*	1
10. Laurel Lake, NY	*I. scapularis*	1
	*A. americanum*	2
	*D. variabilis*	1
11. Tukahoe Hill Preserve, NY	*I. scapularis*	1
12. Fire Island, NY	*A. americanum*	1
13. Giles County, VA	*I. scapularis*	1
14. New Kent County, VA	*I. scapularis*	2
	*A. americanum*	1

a Sites 4 to 12 were all located in Suffolk County, Long Island, NY.

**TABLE 2:** Summary of the ticks analyzed by UHTS

Tick species	No. of pools	No. of ticks
*Ixodes scapularis*	39	879 adults
	10	259 nymphs
*Amblyomma americanum*	34	720 adults
*Dermacentor variabilis*	8	163 adults
Total	91	2,021

**TABLE 3:** Summary of viruses identified by high-throughput sequencing

Virus detected by UHTS^a^	Viral classification	Tick host	Genome length(s) (nt)^b^	Accession no.
Powassan virus	*Flaviviridae* (flavivirus)	*I. scapularis*	10,647^c^	KJ746872, MG647779–MG647783
South Bay virus	*Bunyavirales* (nairovirus-like)	*I. scapularis*	13,892, 5,530	KM048320, KM048321
Blacklegged tick phlebovirus 1	*Bunyavirales* (phlebovirus-like)	*I. scapularis*	6,733, 2,468	KM048313, KM048314
Blacklegged tick phlebovirus 2	*Bunyavirales* (phlebovirus-like)	*I. scapularis*	6,733, 2,445	KM048315, KM048316
**Blacklegged tick phlebovirus 3**	*Bunyavirales* (phlebovirus-like)	*I. scapularis*	6,704, 1,404	KU230449, **KU230450**
**Laurel Lake virus**	*Bunyavirales*	*I. scapularis*	7,253, 1,142, 2,506	**KX774630**, **KX774631**, **MG256515**
Suffolk virus	*Chuviridae*	*I. scapularis*	10,795	NC028243
*I. scapularis***-**associated virus 1	Unclassified	*I. scapularis*	2,801	KM048318
*I. scapularis***-**associated virus 2	Unclassified	*I. scapularis*	2,334	KM048319
***I. scapularis*-associated virus 3**	Unclassified	*I. scapularis*	983	**MG677814**
***I. scapularis*-associated virus 4**	Unclassified	*I. scapularis*	881	**MF962656**
***I. scapularis*-associated virus 5**	*Bunyavirales*	*I. scapularis*	6,526	**MG256513**
***I. scapularis*-associated virus 6**	*Bunyavirales*	*I. scapularis*	7,259	**MG256514**
***I. scapularis* partitivirus**	*Partitiviridae*	*I. scapularis*	242	**MG647778**
**Avian-like circovirus**	*Circoviridae*	*I. scapularis*	1,939	**KU230452**
**Blacklegged tick picorna-like virus 1**	*Picornavirales*	*I. scapularis*	2,180, 648	**MG647769**, **MG647774**
**Blacklegged tick picorna-like virus 2**	*Picornavirales*	*I. scapularis*	1,656, 1,471, 1,133, 1,112	**MG647770**–**MG647773**
**Ilarvirus**	Unclassified	*I. scapularis*	1,975, 606	**MG647776**, **MG647777**
**Blacklegged tick chuvirus 2**	*Chuviridae*	*I. scapularis*	11,518	**MF360789**
**Blacklegged tick rhabdovirus 1**	*Rhabdoviridae*	*I. scapularis*	13,841	**MF360790**
**New Kent County virus**	*Rhabdoviridae*	*I. scapularis*	14,814	**MF615270**

American dog tick phlebovirus	*Bunyaviridae* (phlebovirus-like)	*D. variabilis*	6,600, 1,813	KJ746901, KJ746902
**American dog tick rhabdovirus 1**	*Rhabdoviridae*	*D. variabilis*	12,073	**MF360791**
**American dog tick rhabdovirus 2**	*Rhabdoviridae*	*D. variabilis*	6,452, 2,287	**MF962659**, **MF962661**
Tetravirus-like virus	Unclassified	*D. variabilis*	5,283	KM048322
**American dog tick-associated virus 1**	Unclassified	*D. variabilis*	2,631	**MF962660**
**American dog tick-associated virus 2**	Unclassified	*D. variabilis*	542	**MF962657**
** **				
**Lone star tick chuvirus 1**	Unclassified	*A. americanum*	11,163	**KU230451**
**Lone star tick dicistrovirus**	*Iflaviridae*	*A. americanum*	5,064	**KX774633**
**Lone star tick nodavirus**	*Nodaviridae*	*A. americanum*	2,784, 1,526	**KX774634**, **KX774635**
**Lone star tick-associated virus 1**	Unclassified	*A. americanum*	3,133	**MF962658**
**Lone star tick densovirus 1**	*Parvoviridae*	*A. americanum*	4,436	**KX774632**
**Lone star tick totivirus**	*Totiviridae*	*A. americanum*	8,524	**MG647775**

a Provisional names of novel viruses discovered by UHTS in this study are shown in bold.

b Corresponds to the complete genome, complete segment, or longest contig sequence generated by UHTS.

c Corresponds to GenBank accession no. KJ746872.

### *Bunyavirales*.

**FIG 2:**
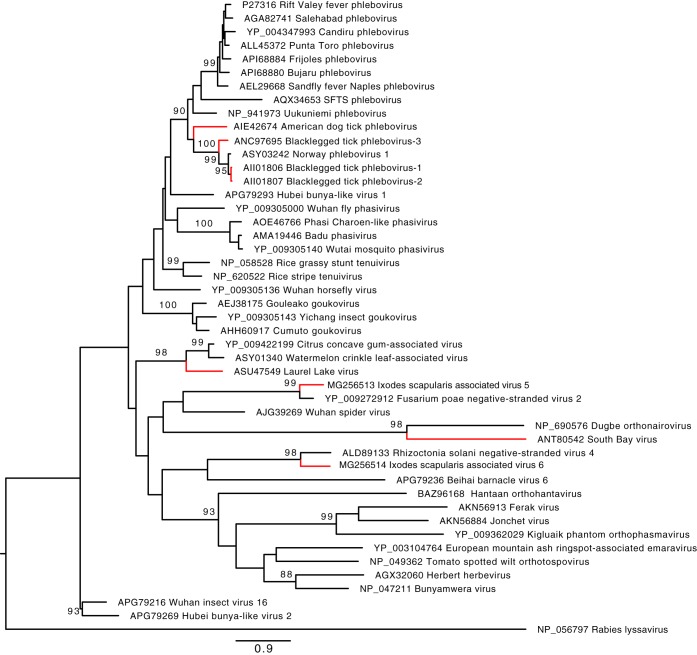
Phylogenetic characterization of the *Bunyavirales*-like viruses identified in this study. Relationships were identified through the alignment of a 386-aa fragment of the RdRp that includes the premotif through motif E of the conserved palm domain. The alignment included the reference sequence of every ICTV-recognized species within the order *Bunyavirales*. Unclassified viruses with sequence homology to the viruses identified in this study were also included. Accession numbers are indicated next to the virus name. Branches representing the viruses identified in this study are shown in red. Rabies virus was used as an outgroup.

*Bunyavirales* is a viral order that consists of multisegmented, negative-sense, single-stranded RNA viruses (15). We identified eight *Bunyavirales*-like viruses—seven in pools of *I. scapularis* (Table 3). In our previous work, we characterized three *Bunyavirales*-like viruses in *I. scapularis* from two different genera: a putative nairovirus, designated South Bay virus (SBV), and two phlebo-like viruses, designated blacklegged tick phleboviruses 1 and 2 (BTPV-1 and -2) (6). All three of these viruses were present in every *I. scapularis* pool examined in this study. We also identified one additional phlebovirus-like virus with sequence similarity to BTPV-1 and -2, provisionally designated blacklegged tick phlebovirus 3 (BTPV-3). We obtained the complete coding sequence for both the L and S segments of this virus. However, as was the case with BTPV-1 and -2, a third M-like segment could not be identified for BTPV-3. The S segment of BTPV-3 contains a 1,404-nucleotide (nt) putative nucleocapsid open reading frame (ORF), whereas the L segment encodes a 6704-nt putative RNA-dependent RNA polymerase (RdRp). The amino acid sequences of BTPV-3 N and L proteins were approximately 30% and 50% similar to the S and L proteins of BTPV-1 and -2. Phylogenetically, BLTPV-3 clusters with BTPV-1, BTPV-2, and Norway phlebovirus 1, a virus recently identified in *Ixodes ricinus* ticks in Europe (12) (Fig. 2). BLTPV-3 sequences were present in 65% of *I. scapularis* pools. We also examined 23 individual ticks from one of the positive pools, with 8 ticks testing positive, indicating this virus has a lower prevalence than BTPV-1 and -2.

Three pools (8%) of *I. scapularis* adults collected at sites 4, 9, and 10 on Long Island (Table 1) were positive for a highly divergent *Bunyavirales*-like virus that we provisionally named Laurel Lake virus (LLV). The genome of LLV contained three segments: a 1.2-kb segment (RNA1) encoding a putative nucleocapsid, a 2.5-kb segment (RNA2) encoding a protein of unknown function, and a 6.6-kb segment (RNA3) encoding the RdRp. Phylogenetic analysis of the amino acid sequence of the RdRp protein revealed LLV forms a monophyletic branch within *Bunyavirales*, along with citrus concave gum-associated virus (CCGV) (GenBank accession no. NC_035759) and watermelon crinkle leaf-associated virus (WCLV) (accession no. KY781187) (Fig. 2) (16, 17). Both of these viruses are plant pathogens that have been recently identified by UHTS. The 254-amino-acid (aa) putative nucleocapsid of LLV is 22% similar and approximately 95 aa shorter than the capsid proteins of CCGV and WCLV. The RdRp is 31% similar. These proteins also have distant homology to the N and L proteins of other viruses within* Bunyavirales*. The 688-aa putative protein encoded by the RNA2 segment is <25% similar to CCGV and WCLV and substantially longer, with a 212-aa N-terminal portion that is not present in these two viruses; however, it does not have homology to proteins from any other viral group. In addition, this protein does not contain any glycoprotein motifs that are typically associated with M segments of bunyaviruses.

We identified two genotypes of LLV by comparing the sequences obtained from the three LLV-positive pools. The nucleotide sequences of the viruses present in two of the pools were 98 to 99% identical in all three segments. The third pool contained a virus that was 96% identical within RNA1, 89% identical within RNA2, and 95% identical within RNA3. To estimate the prevalence of LLV, we used PCR to test the cDNA of 37 adult *I. scapularis* ticks collected in Westchester County, NY, in 2008 and 23 adult *I. scapularis* ticks from New London, CT, collected in 2010. Only two ticks were positive—both from New York. These results, combined with LLV being present in <10% of UHTS pools, suggest this virus is rare in *I. scapularis*.

We also identified sequences of two distinct contigs from *I. scapularis* with homology to the RdRp of newly discovered unclassified viruses that cluster within *Bunyavirales*. We provisionally named these *Ixodes scapularis*-associated viruses 5 and 6 (ISAV-5 and -6). The sequence of ISAV-6 was most similar to *Rhizoctonia solani* negative-stranded virus 4 (GenBank accession no. KP900923), while ISAV-5 clustered with *Fusarium poae* negative-stranded virus 2 (accession no. LC150619) (Fig. 2) (18, 19). The segments encoding the RdRp of both viruses were identified in metagenomic analysis of fungi, although other segments were not identified. ISAV-5 and -6 were both present in the same two *I. scapularis* pools. We subsequently tested the 24 individual ticks that comprised one of the positive pools and detected both viruses in the same two ticks. Our data suggest that both viruses are likely acquired simultaneously by *I. scapularis*.

### *Rhabdoviridae*.

**FIG 3:**
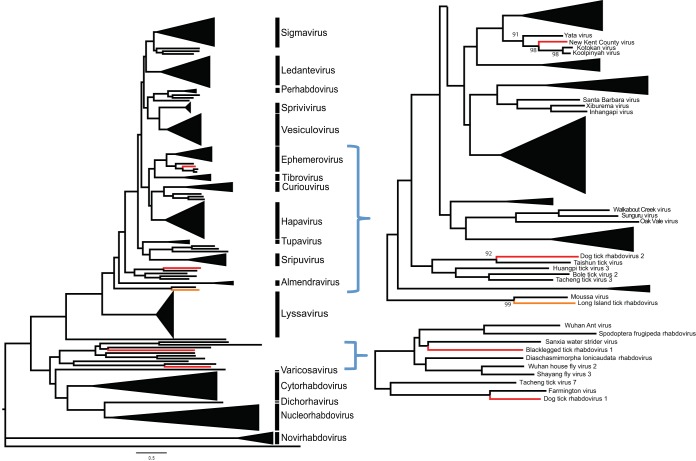
Phylogeny of the novel rhabdoviruses identified by UHTS. The phylogenetic tree displays the relationship of all the currently ICTV-recognized genera and species of the family *Rhabdoviridae*. Branches representing the viruses identified in this study are shown in red. Long Island tick rhabdovirus, a tick virus we identified in our previous work but not in this study, is shown in orange. Also included in the tree are rhabdoviruses not yet approved by ICTV that have similarity to the viruses identified in this study. A zoomed-in portion of the tree with the new viruses is shown on the right. A chuvirus, Wuhan tick virus, was used as an outgroup.

**FIG 4:**
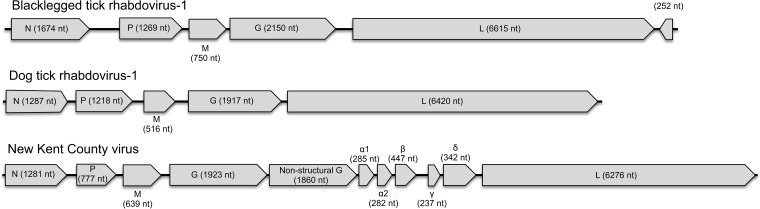
Genome schematics of blacklegged tick rhabdovirus 1, dog tick rhabdovirus 1, and New Kent County virus.

We identified four new viruses with sequence homology to viruses in the family *Rhabdoviridae*: two in *I. scapularis* and two in *D. variabilis* (Table 3). The two *I. scapularis*-borne viruses were provisionally named blacklegged tick rhabdovirus 1 (BLTRV-1) and New Kent County virus (NKCV). BLTRV-1 was present in two pools of adult ticks (one each from Connecticut and New York) and phylogenetically clustered with rhabdoviruses recently identified by UHTS in insects (Fig. 3). We tested 22 individual ticks that comprised one of the BLTRV-1-positive pools by reverse transcription-PCR (RT-PCR), with only one tick testing positive. NKCV was identified in two of the three pools of *I. scapularis* ticks collected in Virginia, both from New Kent County. None of the Connecticut and New York pools were positive for this virus. Analysis of the NKCV genome revealed that it is similar in structure and sequence to rhabdoviruses classified within the genus *Ephemerovirus*. NKCV encodes a nonstructural glycoprotein and 5 accessory proteins upstream of the polymerase, a feature of other ephemeroviruses (Fig. 4). Phylogenetic analysis of the L protein revealed that NKCV clustered with Kotokan, Koolpinyah, and Yata viruses, which make up one of the two clades within *Ephemerovirus* (Fig. 3).

The two rhabdoviruses identified in *D. variabilis* were provisionally named American dog tick rhabodoviruses 1 and 2 (ADTRV-1 and -2). ADTRV-1 was present in two *D. variabilis* pools and was most similar to Farmington virus (FARV), which was isolated from a wild bird in Farmington, CT (20). The L protein of ADTRV-1 had 45% amino acid sequence similarity to that of Farmington virus and <30% similarity to those of other rhabdoviruses. The high sequence divergence of ADTRV-1 and FARV suggests both viruses likely represent a new genus within *Rhabdoviridae*.

ADTRV-2 sequences were present in three out of eight *D. variabilis* pools sequenced. However, due to insufficient sequence coverage, we only assembled the nucleocapsid and the polymerase portions of the genome. Analysis of the amino acid sequence of both proteins revealed that ADTRV-2 sequences were most similar to those of Taishun tick virus and Huangpi tick virus 3, which were identified in ticks from China (Fig. 3). Little is known about this group of viruses, although they likely will also be classified into a distinct genus within *Rhabdoviridae*.

### Chuviruses.

We previously reported the discovery of a virus in *I. scapularis* with homology to *Mononegavirales*-like RdRp. Here, we report the complete sequence of this virus, provisionally named Suffolk virus (SUFV) (accession no. NC028243). Analysis of the amino acid sequence and genome structure indicated that SUFV is similar to a group of recently identified arthropod-associated viruses that were assigned to a provisional family designated *Chuviridae* (7). SUFV was present in *I. scapularis* ticks in all three geographic areas surveyed, including 11 out of 28 pools from Long Island, 17 out of 18 pools from Connecticut, and 2 out of 3 pools in Virginia.

In addition, we identified two other putative chuviruses, designated blacklegged tick chuvirus 2 (BLTCV-2) and lone star tick chuvirus 1 (LSTCV-1). BLTCV-2 was detected in a single *I. scapularis* pool from Virginia. LSTCV-1 was present in >90% of pools from New York and was also present in the one sequenced pool of *A. americanum* from Virginia.

**FIG 5:**
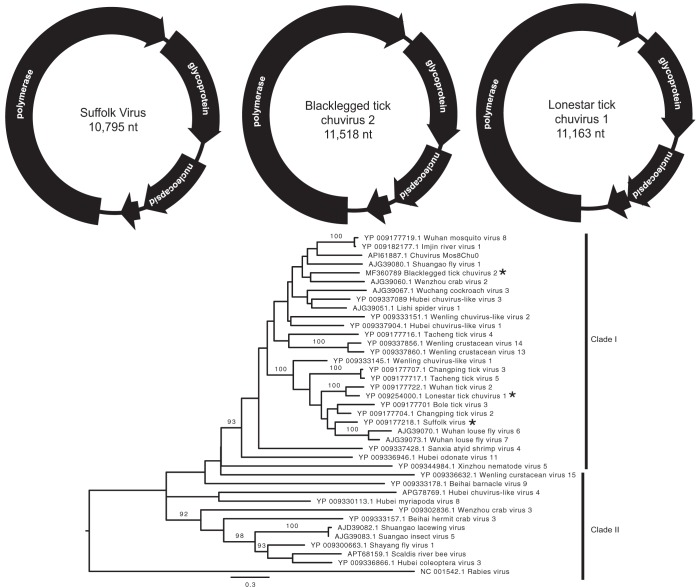
Characterization of chuvirus-like viruses. Panel A shows the genome schematic diagrams of the three novel chuviruses identified in our study. Panel B shows the phylogenetic relationship of these viruses (indicated by stars) to other chuviruses based on a conserved 331-aa fragment of the RdRp. Only nodes with bootstrap values greater than 90 are shown. Clade I represents viruses with circular single-segment genomes, and clade II represents viruses with linear or circular multisegmented genomes. Rabies virus was used as an outgroup.

Analysis of complete genomes revealed all three viruses have a circular genome of approximately 10.8 to 11.5 kb in length that includes four putative ORFs (Fig. 5A). ORFs 1, 2, and 3 encode the RdRp, a putative glycoprotein, and a putative nucleoprotein, respectively. Due to a lack of homology to proteins outside of *Chuviridae*, the potential function of ORF4 is unknown. Phylogenetically, chuviruses cluster into two distinct groups (Fig. 5B). Clade I includes viruses with circular genomes, and clade II contains viruses with linear or circular multisegmented genomes. All three viruses identified in our study cluster within clade I, although they are more closely related to viruses identified in China than to each other.

### *Nodaviridae*.

**FIG 6:**
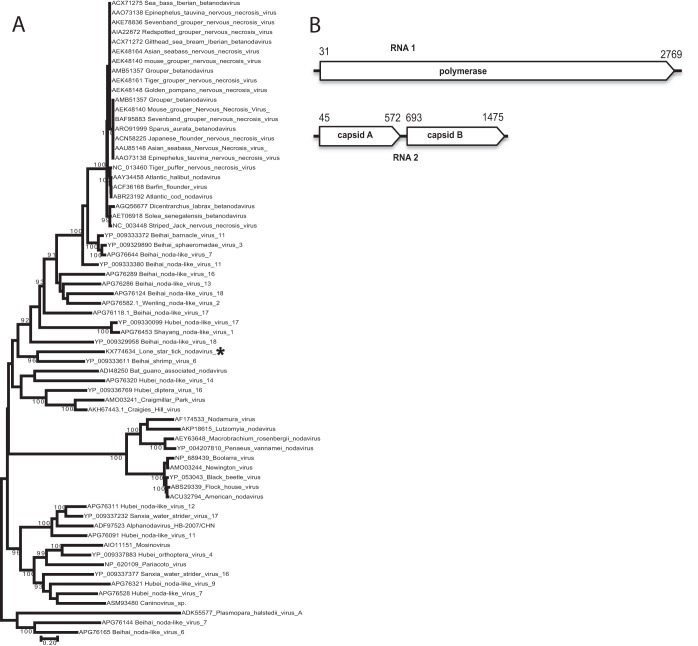
Phylogeny and genome schematic of lone star tick nodavirus. Panel A shows the phylogenetic analysis for 66 representative members of the *Nodaviridae* family over a total of 606 aa positions of the conserved region of the RdRp. The lone star tick nodavirus is indicated with an asterisk. Panel B shows the genome diagram of the two lone star tick nodavirus segments.

A new nodavirus, named lone star tick nodavirus 1 (LSTN-1), was present in three *A. americanum* pools. Nodavirus genomes typically consist of a bipartite, linear, single-stranded positive-sense RNA genome (15). RNA1 encodes the polymerase, and RNA2 typically encodes a polyprotein posttranslationally cleaved into two capsid components. Two genera of nodaviruses are recognized: *Alphanodavirus*, associated with arthropods, and *Betanodavirus*, isolated from fish and other marine animals. Phylogenetically, LSTN-1 clusters with arthropod viruses and is the first nodavirus discovered in ticks (Fig. 6). LSTN-1 contains an atypical structure of RNA2, with two ORFs instead of a single polyprotein. The 528-nt 5′-proximal ORF has limited similarities to viral capsid proteins of nodaviruses, while ORF2 has no similarity to any known protein.

### Sobemo- and Luteo-like viruses.

Recent UHTS studies of invertebrates revealed the existence of a large diversity of Sobemo-like or Luteo-like viruses present across a wide range of arthropod families (7, 10). These viruses typically consist of a 3- to 3.5-kb genome that contains two ORFs. We identified four viruses that have sequence homology to viruses within this group. At least one virus was present in pools from each tick species, with one novel virus in *A. americanum* (lone star tick-associated virus 1), one novel virus in *D. variabilis* (American dog tick-associated virus 1), and two viruses in *I. scapularis* we identified previously (*Ixodes scapularis*-associated viruses 1 and 2) (Table 3).

### Powassan virus.

We detected Powassan virus (POWV) (deer tick virus, lineage II) in 7 out of 37 adult pools. Six POWV-positive pools originated from sites 4, 6, and 7 on Long Island, while one POWV-positive pool of ticks originated from site 1 in Connecticut. All viruses were genetically homogenous (99% nucleotide identity) to the LI-1 strain we identified in 2013. POWV was not present in any of the 10 pools of nymphs or in the three *I. scapularis* pools of adults from Virginia. By PCR, we screened an additional 70 adult ticks from Virginia that were also negative.

### Other viruses.

We detected two viruses (90% amino acid identity) in *I. scapularis* and *D. variabilis* (*Ixodes scapularis*-associated virus 4 and American dog tick-associated virus 2) that were similar to viruses identified in *Drosophila* (Table 3) (21). In lone star ticks, we obtained the complete coding sequence of a new densovirus and totivirus. We also identified several viruses in *I. scapularis* that were present in only a single pool. These included an avian-like circovirus, an Ilarivirus, a partitivirus, and two picorna-like viruses. All five viruses displayed <65% amino acid similarity to the next closest virus within their group.

Since most of the surveyed sites were clustered within New York and Connecticut, we sampled ticks from Virginia to assess variations in the tick virome due to geography. All of the highly prevalent *I. scapularis*-borne viruses were also found in Virginia. However, despite testing only three pools, we identified two viruses (BLTCV-2 and NKCV) that were not present in any of the 46 *I. scapularis* pools from New York and Connecticut. Thus, we expect that exploration of additional sites throughout the range of *I. scapularis* would identify additional viruses.

### Transmission.

We next examined whether vertical transmission from the female ticks to their eggs may account for the high prevalence of SBV, BLTPV-1, and SUFV in *I. scapularis*. cDNA generated from 20 individual replete *I. scapularis* females was tested by RT-PCR for the presence of each virus. We detected a high prevalence (>50%) of each virus in individual ticks, including nine infected with all three viruses (Table 4). We then examined a portion of the egg mass or larvae from four of these ticks for evidence of these viruses. We detected at least one of the viruses in the eggs/larvae from each of the corresponding parental ticks, confirming that these viruses were transovarially transmitted.

**TABLE 4:** PCR analysis of transovarial transmission of SBV, BLTPV-1, and SUFV

Female tick	Amplification in tick^a^			Female tick source of eggs/larvae	Amplification in eggs/larvae^a^		
	SBV	BLTPV-1	SUFV		SBV	BLTPV-1	SUFV
1	+	+	+	1	−	+	+
2	+	−	−	2	+	−	−
3	−	+	−	3	−	+	−
4	+	+	+	4	−	+	−
5	−	+	−	5	ND	ND	ND
6	−	−	−	6	ND	ND	ND
7	−	+	−	7	ND	ND	ND
8	−	+	+	8	ND	ND	ND
9	−	+	−	9	ND	ND	ND
10	−	−	+	10	ND	ND	ND
11	+	+	−	11	ND	ND	ND
12	−	+	+	12	ND	ND	ND
13	+	+	+	13	ND	ND	ND
14	−	+	−	14	ND	ND	ND
15	+	+	+	15	ND	ND	ND
16	+	+	+	16	ND	ND	ND
17	+	+	+	17	ND	ND	ND
18	+	+	+	18	ND	ND	ND
19	+	+	+	19	ND	ND	ND
20	+	+	+	20	ND	ND	ND
Total	11/20	17/20	12/20		1/4	3/4	1/4

a +, positive amplification; −, no amplification; ND, not done.

## DISCUSSION

The increasing incidence of tick-borne diseases presents a major challenge to public health worldwide. Improving our understanding of the diversity of the tick microbiome will help guide the identification of emerging tick-borne agents. The principal aim of our study was to catalogue viruses present in *I. scapularis*, *D. variabilis*, and *A. americanum*, the primary tick species associated with transmission of human pathogens in the eastern part of the United States. Through this work, we sought to gain insight into the virome diversity of these important tick species, in order to assess any potential role of these viruses in the tick life cycle.

Based on our previous findings and UHTS analyses of other arthropods, the high diversity of viruses uncovered in this study was not unexpected (7, 10, 21, 22). Recent virome studies have revealed that arthropods and other invertebrates are hosts to a wide range of highly divergent viruses. Members of some of these viral groups have been found across a large number of different arthropods but have not been detected in vertebrates. Although the roles of these viruses in the arthropod life cycles are largely unknown, they are not likely to be horizontally transmitted to other hosts. Although some of the viruses characterized in this study are phylogenetically related to human or animal pathogens, we currently do not have evidence suggesting their potential for horizontal transmission. We also cannot exclude the possibility that some of the identified viral sequences may represent viruses that do not replicate in the tick itself. Instead, these sequences may originate from viruses parasitizing other hosts present within the tick, such as a fungus or nematode, or may represent remains of a virus ingested during a blood meal.

The majority of the samples analyzed in our study were comprised of *I. scapularis* pools. We focused our work on this tick species because of its role as the principal vector of *Borrelia burgdorferi*, the agent of Lyme disease (23). With an estimated 300,000 annual cases of Lyme disease in the United States, *I. scapularis* has a substantial impact on public health, and coinfections have the potential to modulate the clinical course of acute Lyme disease or posttreatment persistent illness (24, 25). Additionally, sampling success was a factor in the final pooling. At the majority of our collection sites, *I. scapularis* and/or *A. americanum* was abundant, whereas *D. variabilis* was not collected in large numbers or was absent.

A striking feature of the *I. scapularis* virome was the presence of a large diversity of *Bunyavirales*-like sequences. Seven of the eight *Bunyavirales*-like viruses detected in our study were identified in *I. scapularis*. Four of these (BTPV-1, BTPV-2, BTPV-3, and SBV) share common features. Each virus was present in ticks from all three states and was highly prevalent in *I. scapularis*, with >25% of individual adult ticks infected. These viruses appeared to lack the glycoprotein-encoding M segment that prototypical bunyaviruses utilize for cellular attachment. We first reported bunya-like viruses that lacked the M segment in our initial tick virome study, and similar viral sequences have subsequently been identified in a wide array of ticks from Europe and Asia (7, 10, 12). We also found that transovarial transmission may play a role in maintaining these viruses in their tick hosts. Based on these common features, we propose that BTPV-1, BTPV-2, BTPV-3, and SBV are viral symbionts of *I. scapularis*. Ticks are known to host a wide array of symbionts, and the presence of transovarially transmitted bacterial symbionts, including many closely related to human pathogens, has been described in all three of the tick species surveyed here (26). Viral symbionts have recently been suggested to exist in several types of arthropods, including ticks (11). Future studies examining horizontal transmission of these viruses will be necessary to confirm our hypothesis.

Due to the high prevalence of these putative viral symbionts, we identified a much greater number of viruses in *I. scapularis* than *A. americanum*. Despite examining a similar number of adult ticks (839 *I. scapularis* versus 720 *A. americanum* ticks), *I. scapularis* ticks were hosts to three times more viruses than *A. americanum*. We found that most *A. americanum* pools were rarely positive for more than one virus, whereas *I. scapularis* pools had, on average, >4 viruses. The primary contributing factor to this difference was the high prevalence of five *I. scapularis* viruses (SBV, BLTPV-1 and -2, SUFV, and ISAV-1), present in >75% of pools from Connecticut and New York. All five viruses were also present in *I. scapularis* from Virginia.

Our study identified four putative novel members of the family *Rhabdoviridae*, a large group of single-stranded negative-sense RNA viruses (15). Rhabdoviruses infect a broad range of vertebrates, invertebrates, and plants (27). Arthropods are hosts to a wide variety of rhabdoviruses and are known vectors for several pathogenic vertebrate viruses. Based on sequence identity and genome structure, only one of the four viruses identified in our study, the *I. scapularis*-borne NKCV, can be grouped into an International Committee on Taxonomy of Viruses (ICTV)-recognized genus, *Ephemerovirus*. Eight other ICTV-recognized ephemerovirus species are recognized. These viruses have been isolated from mosquitos, biting midges, or ruminants from subtropical and tropical regions of Africa, Australia, and Asia (27). Thus, NKCV represents the first tick-associated ephemerovirus and the first to be discovered in North America. Although ephemeroviruses have not been associated with human disease, Kontonkan and bovine fever emphemeroviruses have been implicated in bovine disease in Africa (28, 29).

The phylogeny of the remaining three viruses is less clear. DTRV-2 and BLTR-2 are both similar to a wide array of viruses identified through UHTS of diverse arthropod species, and little can be discerned about their potential for transmission. DTRV-1 forms a unique cluster with Farmington virus, a virus capable of infecting mammalian cells that was isolated from an unidentified wild bird in Connecticut in 1969 (20). Further studies of DRTV-1, including determination of host tropism, may help clarify if this virus presents a risk for human infection.

POWV is currently the only tick-borne virus associated with human disease circulating in the northeastern United States. POWV was present in <10% of *I. scapularis* pools, in agreement with surveillance studies that typically show low prevalence of POWV (<5%) in individual ticks (30–33). We detected POWV in pools from both New York and Connecticut, but not in ticks from Virginia. To our knowledge, the virus has not been shown to be present in the southern United States.

In summary, we performed a comprehensive UHTS analysis of tick viromes that substantially expands the current knowledge of viruses associated with *I. scapularis*, *D. variabilis*, and *A. americanum*. The identification and characterization of these viruses will enable future work focused at determining the role of these viruses in the tick life cycle.

## MATERIALS AND METHODS

### Tick collections and nucleic acid extractions.

Ticks (*I. scapularis*, *D. variabilis*, and *A. americanum*) were collected in 2015 and 2016 at 14 sites in New York, Connecticut, and Virginia (Fig. 1; Table 1). Specimens were kept frozen at −80°C until processing. All tubes, wash reagents, beads, and media were sterilized by UV radiation prior to processing.

Ticks were sorted by species, pooled (mean *n =* 22 ticks per pool) (Table 1), and washed (once with 70% ethanol and three times with H_2_O). Each pool was homogenized using the TissueLyser II (Qiagen) in 1 ml of viral transport medium (VTM) with four 5-mm stainless steel beads in an Eppendorf Safe-Lock tube. Homogenates were centrifuged at 8,000 rpm for 1 min to precipitate cellular debris; the supernatant was removed and then purified through a 0.45-μm-pore filter (Millipore). Nonencapsulated nucleic acids were digested with RNase A (15 min at room temperature) followed by treatment with Turbo DNase and Benzonase (30 min at room temperature). Total nucleic acids (TNAs) from 300 μl of nuclease-treated homogenate were extracted using the easyMAG extraction platform (BioMérieux) and eluted in 40 μl, with 11 μl used in a reverse transcriptase reaction (Superscript III; Invitrogen) prior to library preparation. For six *I. scapularis* adult pools, we used a modified method for homogenization, in which individual ticks (*n =* 20) were washed as described above and individually homogenized in 50 μl of VTM. Twenty microliters of the homogenate from each tick was pooled, and the entire pooled homogenate was filtered and nuclease treated as described above. TNAs from 300 μl of the pooled homogenate were extracted and eluted in 40 μl. The remaining 30 μl of homogenates from individual ticks were extracted individually and used for single-agent reverse transcription-PCR (RT-PCR).

### Library preparation.

Double-stranded DNA was mechanically sheared to an average length of 200 nt and purified. Sequencing was performed on the Illumina HiSeq 4000 system (Illumina, San Diego, CA) using the Hyper Prep kit (KAPA Biosystems, Boston, MA). The demultiplexed FastQ files were adapter trimmed using the cutadapt program (v1.8.3) (34). Adapter trimming was followed by generation of quality reports using FastQC software (v0.11.5), which were used to determine filtering criteria based on the average quality scores of the reads, presence of indeterminate nucleotides, and homopolymeric reads (35). The reads were quality filtered and end trimmed with PRINSEQ software (v0.20.3) (36). Host background levels were determined by mapping filtered reads against a tick reference database using Bowtie2 mapper (v2.2.9) (37). The host-subtracted reads were *de novo* assembled using the MIRA (4.0) assembler (38). Contigs and unique singletons were subjected to homology search using Megablast against the GenBank nucleotide database. Sequences that showed low or no homology at the nucleotide level were subjected to a BLASTX homology search against the viral GenBank protein database. Sequences from viral BLASTX analysis were subjected to a second round of BLASTX homology search against the complete GenBank protein database to correct for biased E values and taxonomic misassignments. For some viruses present at a low abundance, we only obtained interspersed reads and no contigs. We then used RT-PCR on cDNA from the virus-positive pool to fill in gaps in the sequence and to generate long genomic fragments. We also employed PCR to confirm that the novel viral RNA sequences originated from authentic viral nucleic acid and not endogenous viral elements. For all authentic viral RNA sequences, amplification products were obtained with RT-PCR, but not with PCR.

### Analysis of transovarial transmission.

We received 20 *I. scapularis* adult female ticks that previously fed to repletion on a rabbit. Engorged females were placed in a single individual tube, maintained at 21 ± 2°C and >90% relative humidity, and allowed to oviposit. When completed, the individual ticks were removed and placed in 1.5-ml Eppendorf tubes, homogenized, and TNAs were extracted from each tick. After cDNA synthesis, each sample was tested for the presence of different viruses by RT-PCR. A portion of egg mass from each tick was placed into a separate 1.5-ml Eppendorf tube. TNAs from the combination of the egg mass and newly hatched larvae were extracted, and cDNA was synthesized and tested by RT-PCR for the presence of the same viruses as the female ticks to provide evidence for vertical transmission.

### Phylogenetic analysis.

All alignments were performed using MUSCLE alignment software in Geneious 10.1.3 (39). Phylogenetic trees were constructed with MEGA 6.06, and the robustness of each node was determined using 1,000 bootstrap replicates (40). For rhabdoviruses, amino acid sequences of the complete RdRp from 157 viruses were aligned, and the phylogenetic relationship was determined using the maximum likelihood (ML) method employing an LG + G model with nearest-neighbor interchange (NNI). For chuviruses, phylogenetic analysis was performed on a 331-aa conserved RdRp fragment from 38 chuvirus-like amino acid sequences, using the ML method employing an LG + G + I model with NNI. For bunyaviruses, phylogenetic analysis was performed on a 386-aa RdRp fragment consisting of the premotif through motif E from 46 viruses within *Bunyavirales*. The phylogenetic relationship was determined using the ML method employing an LG + G + F model with NNI. For nodaviruses, a 606-aa fragment of the conserved region of the RdRp from 66 putative nodaviruses was used to generate a maximum likelihood tree with the Whelan and Goldman model with discrete gamma distribution (+G) and using the nondefault amino acid frequencies (+F) of the model.

### Accession number(s).

Accession numbers newly reported here are shown in bold in Table 3.
